# Sinus of valsalva aneurysm in Blau's syndrome

**DOI:** 10.1186/1749-8090-5-16

**Published:** 2010-03-26

**Authors:** Faisal Mourad, Augustine Tang

**Affiliations:** 1 Lancashire Cardiac Center, Blackpool Victoria Hospital, Blackpool, Lancashire, UK

## Abstract

Blau syndrome is a rare granulomatous disorder inherited in an autosomal dominant manner characterized by the early appearance of granulomatous arthritis, skin rash and anterior uveitis. There are very few data on the cardiovascular manifestations of Blau syndrome. Here we report the first case of sinus of valsava aneurysm in Blau syndrome. In isolated unruptured aneurysms of a sinus of Valsalva without compromise of the aortic valve and/or the coronary ostia, repair may be accomplished by simple placation of the aneurysm or excision of the aneurysm(s) and patch closure of the defect(s) between the aortic annulus and the sinu-vascular ridge. Because of the particular conditions in our case, the repair was performed with replacement of the aortic valve and root using a composite graft employing a modified Bentall's technique.

## Case Report

A 37 year old lady with Blau syndrome was being followed up by transthoracic echocardiogram for mild to moderate mitral valve prolapse was referred to us after accidental discovery of an echogenic mass associated with the aortic root and right atrium. She had been complaining of slight shortness of breath complying with NYHA grade I.

Past medical history included congenital bilateral ptosis, De Quervain's thyroiditis, congenital bilateral hearing problems. Apart from her cardiac manifestations the clinical picture was consistent with Blau's Syndrome as suggested by The Clinical Genetics Department in St. Mary's hospital, Manchester. Her older sister died as a neonate shortly after birth from complex congenital heart disease, although no further detail was available.

On examination she was found to have saddle nose deformity and camptodactyly. She also has a strikingly erythematous rash affecting her thighs, lower legs, flexures of forearms and sparing the knee flexures and popliteal fossa. Previously operated upon hammer toes and forefoot deformity. Marked contractures affecting the fingers in both her hands. Precordial examination revealed a mild systolic murmur audible over the apex. Specifically there was no diastolic murmur. There was no evidence of heart failure.

## Investigations

**Figure 1:**
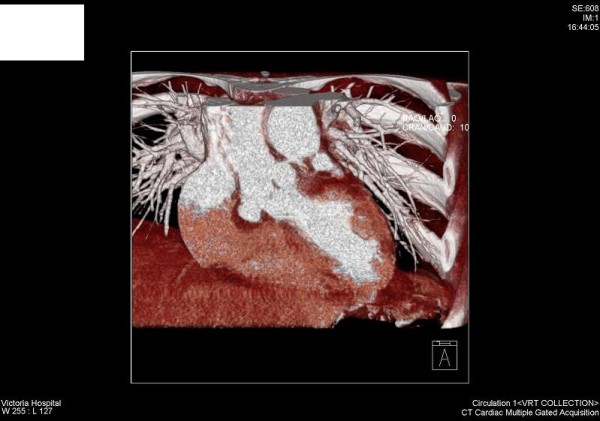
**CT scan showing: non coronary cusp aneurysm with intramural thrombus**.

Chest x-ray showed cardiomegaly with a slightly widened mediastinum. The ECG confirmed sinus rhythm with no conduction abnormalities. The transoesophageal echo showed the presence of an aneurysm of the right and non coronary sinus of Valsalva. There was a suggestion of either a thrombus within the sinuses or an intramural haematoma. The native tricuspid aortic valve appeared to be functioning well without any evidence of stenosis or significant regurgitation. Left ventricular systolic function was preserved. CT of the aortic root demonstrated an aneurysm of the sinus of Valsalva. The non-coronary sinus extended into the right atrium with what appeared to be a thrombus at the base Fig (1). Cardiac MRI confirmed the presence of an aneurysm of the right and non-coronary sinus although the morphology and dimensions of the ascending aorta and the aortic arch appeared normal. Coronary angiography revealed normal coronary anatomy.

During surgical pre-assessment cold agglutinin antibodies were identified in her blood. After serial testing these were found to be activated at room temperature but with minimal activity at 30 degrees or 37 degrees. Extensive discussion and counselling prior to surgery with the patient and her husband took place. She ultimately decided to go for a mechanical prosthesis should a limited repair or a David procedure not be feasible.

## Surgery

**Figure 2:**
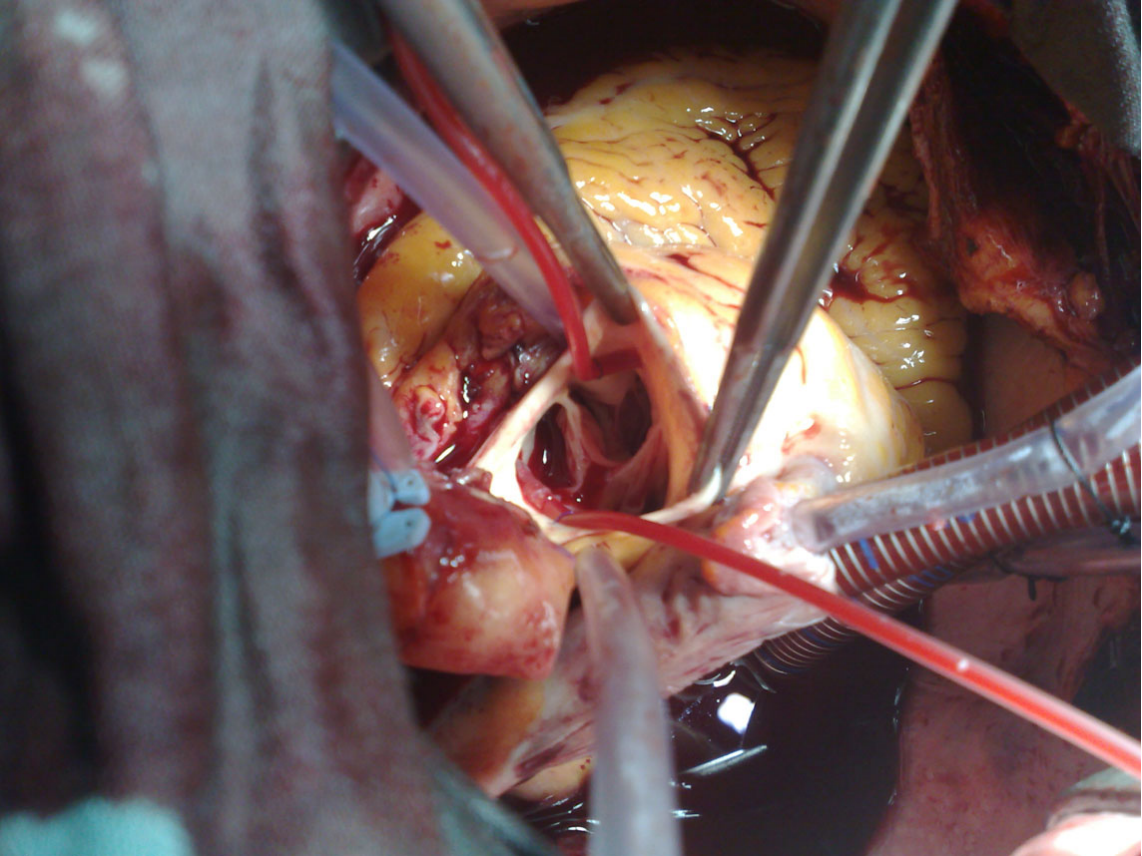
**Sinus aneurysm with aortic sinus trabeculations**.

On external inspection of the aortic root, no apparent aneurysm is visible, suggesting therefore that most of the aneurysmal dilatation occurs hidden under the right ventricular outflow tract and the adjacent part of the right atrium Fig (2).

Following systemic Heparinisation, cardiopulmonary bypass was instituted using bicaval venous drainage (32 gauge) and ascending aortic inflow (24 gauge). Once on full flow the perfusion pressure was maintained >65 mmHg and the core temperature cooled to 32 degrees, but not below because of the cold agglutinins. Following discussion with a number of experienced aortic root surgeons it was decided to proceed with warm blood cardioplegia at 32 degrees.

**Figure 3:**
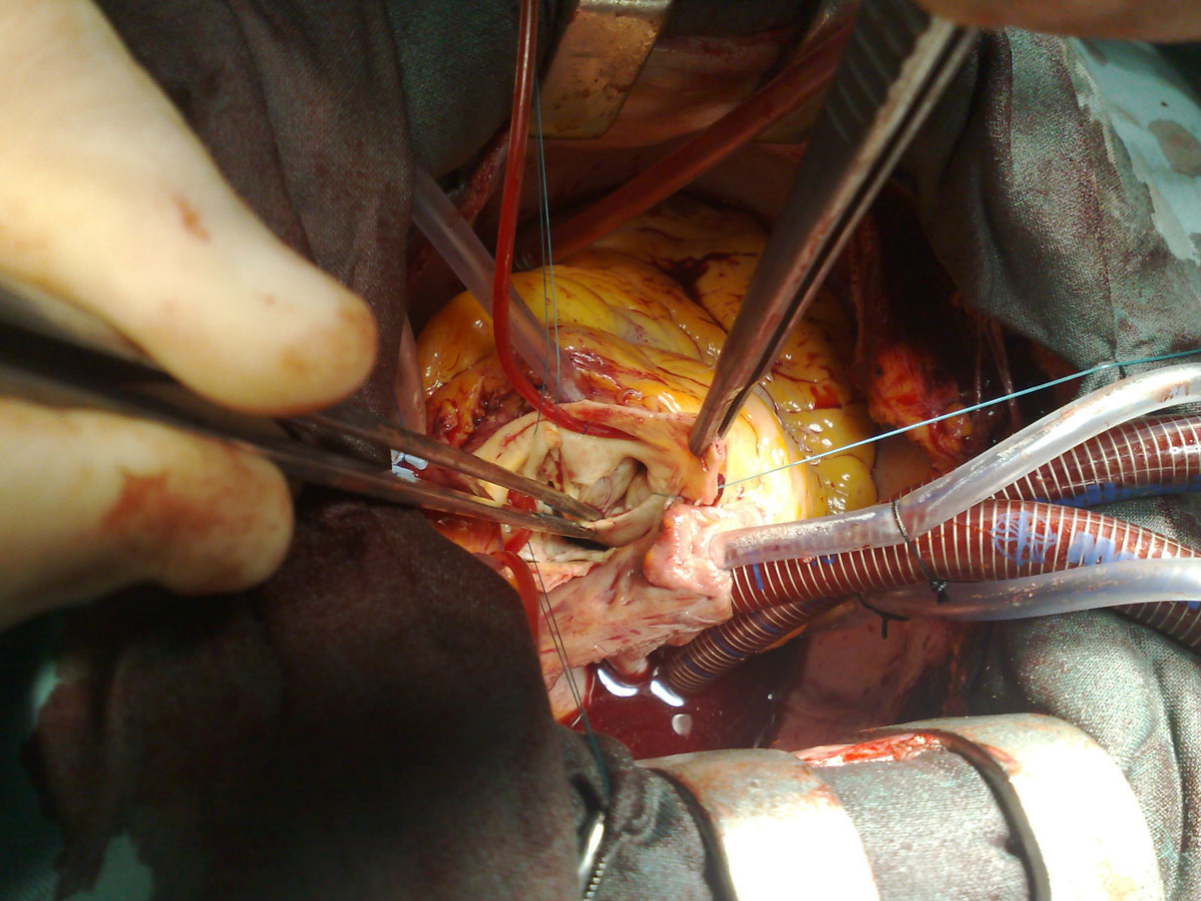
**Sinus aneurysm**.

Careful inspection of the aortic root showed a highly dysplastic arrangement of the leaflets in relation to the aortic sinuses. There was extensive trabeculation along the insertion lines where the leaflets join the sinus walls, to the extent that no trace of normal tissue is seen in this area. In both the right and the non-coronary sinuses there was a large saccular aneurysm lined with mural thrombus Fig. (2, 3). The aortic sinuses were excised along with most of the saccular aneurysm wall and the laminated thrombus. A 23 mm Carbo-Seal composite mechanical prosthesis was selected. This was secured to the native annulus using 3 running 2/0 Prolene sutures. Left and right coronary buttons were anastmosed to the composite graft.

## Post operative complications

There were episodes of haemodynamic instability in the same day of surgery. A TOE performed showed a 1 cm global pericardial fluid collection. There were signs of possible compression of the left atrium. It was decided that an emergency re-sternotomy would be warranted.

Following re-sternotomy and evacuation of blood clots, the patient had smooth post operative recovery and was discharged home in a stable condition 12 days after surgery.

## Follow up

Eight weeks after surgery, she continued to be in good condition on follow up examination with no change to the mitral valve by transthoracic echocardiography.

Histolopathological examination of the aneurysms revealed a non-specific myxomatous degeneration. Aortic leaflets had shown chronic non specific inflammatory reaction.

## Discussion

Blau syndrome is a rare granulomatous disorder inherited in an autosomal dominant manner characterized by the early appearance of granulomatous arthritis, skin rash and anterior uveitis [1]. It was described by Edward Blau in 1985, the same year in which Douglas Jabs reported a very similar family. Clinically indistinguishable from early onset sarcoidosis (EOS), both are now known to share a mutated form of caspase recruitment domain-15 (CARD 15), a protein involved in activation of nuclear factor kappa B which is in turn an up-regulator of pro-inflammatory cytokine transcription. An association between BS and EOS was suspected for years given the striking similarities of the core triad (arthritis-uveitis-dermatitis) and a common emerging pattern of systemic involvement. Hence, the familial form (BS) and the sporadic form (EOS) are almost certainly the same illness/defect, inherited in the first and acquired in the second as a result in most cases of a de novo mutation[2]. There are very few data on the cardiovascular manifestations of Blau syndrome. Here we report the first case of sinus of valsava aneurysm in Blau syndrome. We believe that this is some form autoimmune aortitis, but further evidence is needed.

Sinus of Valsalva aneurysms occur infrequently in the general population, with an incidence of approximately 0.1% [3]. The majority are congenital, due to a defect in aortic media, but they may also follow pathologies including atherosclerosis, trauma, endocarditis and syphilis [4]. In a series of 129 patients [3], 52% of aneurysms arose from the right coronary sinus, 34% from the non-coronary sinus and 14% from the left coronary sinus. Most are clinically silent until they rupture, but enlarging aneurysms can lead to right ventricular outflow tract obstruction, coronary artery compression, complete heart block, and they are a potential source of cerebro-vascular emboli [4]. Symptoms are non-specific and include dyspnoea, fatigue, chest pain and palpitations [3]. Rupture typically occurs into the right ventricle (or right atrium for non-coronary sinus aneurysms) and without surgery carries a poor prognosis [5]. Operative procedures include simple plication, patch repair, aortic root replacement, and aortic valve replacement/repair. Prosthetic aortic root replacement and re-implantation of the coronary arteries is performed in patients with a dilated annulus and multiple sinus involvement, where simple plication or patch repair is not possible.

In isolated unruptured aneurysms of a sinus of Valsalva without compromise of the aortic valve and/or the coronary ostia, repair may be accomplished by simple placation of the aneurysm or excision of the aneurysm(s) and patch closure of the defect(s) between the aortic annulus and the sinu-vascular ridge [6,7]. In cases with additional aortic valve defects and/or if the right or left coronary ostium originates from the aneurysmal area, a more complex repair seems advisable in order to avoid subsequent complications. Because of the particular conditions in our case, the repair was performed with replacement of the aortic valve and root using a composite graft employing a modified Bentall's technique [8].

## Competing interests

The authors declare that they have no competing interests.

## Authors' contributions

FM - editor and was first assistant in the sugical procedure. AT - operating surgeon and revised the text. All authors read and approved the final manuscript.
